# Real-world comparison of avelumab maintenance versus pembrolizumab at progression after first-line platinum-based chemotherapy in metastatic urothelial cancer

**DOI:** 10.1016/j.esmorw.2026.100685

**Published:** 2026-02-16

**Authors:** J. Bosveld, B.B.M. Suelmann, M.J. Bijlsma, M.D. Franken, K.K.H. Aben, ProBCI Study Group, R.P. Meijer, A. Richters

**Affiliations:** 1 Department of Oncological Urology, University Medical Center Utrecht, Utrecht, The Netherlands; 2 Department of Research & Development, The Netherlands Comprehensive Cancer Organisation, Utrecht, The Netherlands; 3 Department of Medical Oncology, University Medical Center Utrecht, Utrecht, The Netherlands; 4 Unit of Pharmacotherapy, Epidemiology and Economics, Groningen Research Institute of Pharmacy, University of Groningen, Groningen, The Netherlands; 5 Department of Medical Oncology, Radboud University Medical Center, Nijmegen, The Netherlands; 6 Science Department IQ Health, Radboud University Medical Center, Nijmegen, The Netherlands

**Keywords:** avelumab maintenance, bladder cancer, immunotherapy, overall survival, pembrolizumab, urothelial cancer

## Abstract

**Background:**

For patients with metastatic urothelial cancer of the bladder (mUCB), the standard of care after first-line (1L) platinum-based chemotherapy has shifted from immune checkpoint inhibitor (ICI) treatment upon progression to maintenance ICI, without a direct comparison between both treatment strategies. We aimed to estimate the effect of the introduction of maintenance avelumab on overall survival (OS) compared with pembrolizumab at disease progression.

**Materials and methods:**

This nationwide cohort study included patients with synchronous mUCB between November 2017 and December 2023 without disease progression after four or more cycles of 1L platinum-based chemotherapy from the Netherlands Cancer Registry. Patients were grouped into the pembrolizumab era or avelumab era representing the prevailing standards of care based on the date of completing chemotherapy. Kaplan–Meier methods and multivariable flexible parametric survival models were used to compare OS, summarized as restricted mean survival time (RMST).

**Results:**

We identified 308 mUCB patients, 153 in the pembrolizumab era and 155 in the avelumab era. Baseline characteristics were mostly comparable between the two groups. ICI use was 56.2% in the pembrolizumab era and 72.3% in the avelumab era (*P* = 0.05). Adjusted RMST difference (avelumab era – pembrolizumab era) up to 24 months was 0.7 months (95% confidence interval –1.1 to 2.3).

**Conclusion:**

No improved survival was observed following the change in standard of care from pembrolizumab at progression to avelumab maintenance, though more patients received ICI. These findings suggest that a treatment-free interval after chemotherapy, followed by ICI at progression, may be as effective as immediate maintenance ICI therapy, while minimizing toxicity, treatment burden, and healthcare resource use.

## Highlights

• This study used real-world data from a population-based nationwide cohort.
• Patients who concluded chemotherapy with stable disease or response were included.
• Change to avelumab maintenance as standard of care did not improve OS.
• Immunotherapy use was higher in the avelumab era.
• The guidelines committee could consider a more balanced recommendation.

## Introduction

Globally, ∼610 000 patients are diagnosed with bladder cancer annually. About 25% present with muscle-invasive disease, and 10%-15% of these cases have metastases.^1^^,^^2^ The standard first-line (1L) treatment for metastatic urothelial cancer (mUC) has been platinum-based chemotherapy (with cisplatin or carboplatin) since the 1980s.^3^ In 2017, second-line (2L) treatment with programmed cell death protein 1 (PD-1) inhibitor pembrolizumab became standard of care for patients with disease progression during or following 1L platinum-based chemotherapy, based on KEYNOTE-045.^4^ Subsequently, the JAVELIN Bladder 100 trial showed that programmed death-ligand 1 (PD-L1) inhibition with maintenance avelumab and best supportive care directly after chemotherapy prolonged overall survival (OS) compared with best supportive care alone in patients with mUC who did not progress after at least four cycles of platinum-based chemotherapy.^5^ This led to its approval by the European Medicines Agency (EMA) in 2020.^6^

Despite the absence of a randomized head-to-head comparison, avelumab maintenance therapy has become the standard of care for this patient population, replacing pembrolizumab upon progression. This shift has moved immune checkpoint inhibitor (ICI) application to an earlier setting, potentially increasing the proportion of patients with ICI exposure and eliminating a treatment-free interval after chemotherapy.

Several cohort studies have evaluated the treatment strategies of immediate ICI with avelumab after chemotherapy versus delayed ICI with pembrolizumab upon progression. However, these studies were compromised by selection bias and limited size (<175 patients).^7^, ^8^, ^9^, ^10^ To ensure a valid comparison between both treatment strategies, patients who are eligible for subsequent ICI should be followed from the point of completing platinum-based chemotherapy, reflecting the clinical decision-making moment for subsequent treatment strategy. Excluding patients who were at that point eligible for delayed pembrolizumab but died before its initiation introduces immortal time bias and leads to an overestimation of the benefit of that strategy. Previous studies that included only patients who actually received ICI are therefore subject to this bias. Instead, using the periods in which either strategy was used as standard of care allows estimation of a population-level survival effect of the standard of care shift.

In the Netherlands, pembrolizumab became generally available in November 2017, followed by avelumab in January 2022 after a positive reimbursement decision, effectively shifting the nationwide standard of care from 2L pembrolizumab to maintenance avelumab. The aim of our study was to estimate the effect of the introduction of avelumab maintenance as standard of care on OS compared with pembrolizumab at progression in patients with mUC of the bladder (mUCB) without disease progression after 1L platinum-based chemotherapy, using nationwide real-world data. We also assessed whether the overall application of ICI changed.

## Materials and methods

### Cohort

For this nationwide cohort study, we identified all patients with synchronous mUCB between 1 November 2017 and 31 December 2023 from the Netherlands Cancer Registry (NCR).^11^ We included all patients without disease progression after at least four cycles of 1L platinum-based chemotherapy, regardless of which subsequent treatments they underwent (more details in Supplementary Figure S1, available at https://doi.org/10.1016/j.esmorw.2026.100685).

Patients who completed 1L chemotherapy before January 2022 were grouped in the pembrolizumab era during which pembrolizumab at progression was standard of care. Patients completing 1L chemotherapy from January 2022 onward were grouped into the avelumab era, when standard of care was maintenance avelumab.^12^ This study was approved by the Privacy Review Board of the NCR (reference number K25.947).

### Clinical data, definitions, and outcomes

The NCR contains data of all patients diagnosed with cancer in the Netherlands since 1989. The registry includes detailed information on patient and tumor characteristics, initial treatment, and vital status, collected by specialized data managers from all hospitals in the Netherlands. The Prospective Bladder Cancer Infrastructure (ProBCI), embedded within the NCR, offers additional clinical data for patients with bladder cancer including performance status, laboratory values, and comprehensive details on all treatments administered at diagnosis and during follow-up. These data were used for this study.^13^ All patients in the NCR are annually linked to the Dutch personal records database to obtain updated vital status information. Vital status information was current as of 31 January 2025.

All baseline patient and disease characteristics were determined directly before chemotherapy. 1L chemotherapy was categorized as cisplatin-based [gemcitabine-cisplatin and (dose-dense) methotrexate, vinblastine, doxorubicin and cisplatin], carboplatin-based (carboplatin-gemcitabine), or a switch from a cisplatin-based to a carboplatin-based regimen. Response to chemotherapy was categorized according to RECIST criteria if reported as such in the electronic patient file,^14^ otherwise treatment response was categorized as either stable disease or response.

Patients treated with avelumab received 800-mg infusions every 2 weeks (Q2W). Patients treated with pembrolizumab received 200 mg every 3 weeks (Q3W) or 400 mg every 6 weeks (Q6W). From July 2021, pembrolizumab dosing followed the Dutch national dose banding guidelines taking into account the patient’s body weight as recommended by the Nederlandse Vereniging voor Medische Oncologie (NVMO).^15^

The outcome was OS, calculated as time from completion of 1L chemotherapy to death. Patients were censored if they were still alive 2 years after completing chemotherapy or at the date of the last vital status linkage (31 January 2025) whichever came first.

### Statistical analysis

Baseline and treatment characteristics were described with frequencies and percentages or medians with interquartile ranges (IQRs). Standardized differences between binary variables were calculated using the difference in proportions divided by the pooled standard deviation. For categorical variables with more than two categories, a Mahalanobis distance approach based on the group-specific proportions was used. The Kaplan–Meier method was used to describe unadjusted 2-year OS stratified by group (pembrolizumab era or avelumab era).

To compare OS between both groups, we fitted a fully parametric flexible survival model adjusted for sex, age, performance status, hemoglobin (dichotomized at <10 g/dl), 1L chemotherapy regimen and response, and metastatic sites (liver, bone, lung, lymph node, lymph node only, and other). In addition, an interaction term between time and group was included to account for nonproportional hazards, because both groups received subsequent ICI treatment to varying degrees and at different time points during follow-up.

The estimated effect of standard of care ICI strategy on OS was summarized as the difference in marginal (population-averaged) restricted mean survival time (RMST) up to 24 months, as an absolute measure of effect. RMST reflects the area under the survival curve up to a specified time horizon (tau), providing an absolute and clinically interpretable measure of survival time difference, and does not assume proportionality of hazards.^16^, ^17^, ^18^ In addition to this primary estimate we calculated a conditional time-varying hazard ratio (HR), a commonly used and interpretable outcome measure that provides a relative effect measure while also relaxing the proportional hazards assumption. Confidence intervals (CIs) were calculated using a nonparametric bootstrap with 500 iterations.

## Results

### Cohort

Between November 2017 and December 2023, 1859 patients were diagnosed with synchronous mUCB in the Netherlands of whom 710 patients received 1L platinum-based chemotherapy. A total of 308 patients were progression-free after receiving at least four cycles of chemotherapy, constituting the study cohort. Of these, 153 completed chemotherapy during the pembrolizumab era, and 155 during the avelumab era (Supplementary Figure S1, available at https://doi.org/10.1016/j.esmorw.2026.100685).

Patient and tumor characteristics stratified by ICI era are summarized in Table 1. Baseline characteristics were mostly comparable between both groups (standardized difference <0.2). Patients in the pembrolizumab era had a slightly higher proportion of metastases in distant lymph nodes than patients in the avelumab era (72.5% versus 60.6%), but the proportion with only distant lymph node metastases was comparable (standardized difference 0.143).

**Table 1:** Baseline characteristics for patients with synchronous metastatic bladder cancer without disease progression after treatment with at least four cycles of platinum-based first-line chemotherapy

Characteristics	Total	Period of completing chemotherapy		Standardized difference
		Pembrolizumab era	Avelumab era	
**Total, *n* (%)**	308 (100)	153 (100)	155 (100)	—
**Sex, *n* (%)**				0.023
Male	223 (72.4)	110 (71.9)	113 (72.9)	—
Female	85 (27.6)	43 (28.1)	42 (27.1)	—
**Age at diagnosis, years**				0.179
0-60, *n* (%)	73 (23.7)	42 (27.5)	31 (20.0)	—
61-70, *n* (%)	106 (34.4)	49 (32.0)	57 (36.8)	—
71-80, *n* (%)	123 (39.9)	59 (38.6)	64 (41.3)	—
≥81, *n* (%)	6 (1.9)	3 (2.0)	3 (1.9)	—
Median, IQR	69 (61-74)	68 (60-74)	69 (62-74)	—
**Performance status**^a^**, *n* (%)**				0.194
ECOG 0	137 (60.4)	78 (63.4)	59 (56.7)	—
ECOG 1	78 (34.4)	40 (32.5)	38 (36.5)	—
ECOG 2	11 (4.8)	5 (4.1)	6 (5.8)	—
ECOG 3-4	1 (0.4)	—	1 (1.0)	—
Unknown	81 (—)	30 (—)	51 (—)	—
**Charlson comorbidity index, *n* (%)**				0.056
0	179 (58.1)	90 (58.8)	89 (57.4)	—
1	61 (19.8)	31 (20.3)	30 (19.4)	—
≥2	68 (22.1)	32 (20.9)	36 (23.2)	—
**Hemoglobin (g/dl)**^a^^,^^b^**, *n* (%)**				0.062
<10	25 (8.9)	11 (8.0)	14 (9.8)	—
≥10	255 (91.1)	126 (92.0)	129 (90.2)	—
Unknown	28 (—)	16 (—)	12 (—)	—
**Renal function (ml/min/1.73 m^2^)**^a^**, *n* (%)**				0.136
<30	3 (1.1)	2 (1.5)	1 (0.7)	—
30-60	94 (33.7)	49 (36.3)	45 (31.3)	—
≥60	182 (65.2)	84 (62.2)	98 (68.1)	—
Unknown	29 (—)	18 (—)	11 (—)	—
**Lactate dehydrogenase (U/l)**^a^**, *n* (%)**				0.026
<250	197 (78.8)	96 (79.3)	101 (78.3)	—
≥250	53 (21.2)	25 (20.7)	28 (21.7)	—
Unknown	58 (—)	32 (—)	26 (—)	—
Median, IQR	203 (172-241)	198 (168-241)	205 (177-241)	—
**Metastatic sites**^c^**, *n* (%)**				
Bone	84 (27.3)	39 (25.5)	45 (29.0)	0.080
Liver	30 (9.7)	14 (9.2)	16 (10.3)	0.040
Lung	69 (22.4)	34 (22.2)	35 (22.6)	0.009
Lymph node	205 (66.6)	111 (72.5)	94 (60.6)	0.254
Lymph node only	138 (44.8)	74 (48.4)	64 (41.3)	0.143
Other	28 (9.1)	13 (8.5)	15 (9.7)	0.041
**First-line chemotherapy, *n* (%)**				0.039
Cisplatin-based	124 (40.3)	63 (41.2)	61 (39.4)	—
Carboplatin-based	161 (52.3)	79 (51.6)	82 (52.9)	—
Gemcitabine-cisplatin switched to gemcitabine-carboplatin^d^	23 (7.5)	11 (7.2)	12 (7.7)	—
**Cycles of chemotherapy, *n* (%)**				0.193
4	48 (15.6)	20 (13.1)	28 (18.1)	—
5	39 (12.7)	17 (11.1)	22 (14.2)	—
6	216 (70.1)	114 (74.5)	102 (65.8)	—
>6	5 (1.6)	2 (1.3)	3 (1.9)	—
**Response to first-line chemotherapy (RECIST/clinical), *n* (%)**				0.176
Response				—
Complete response	13 (4.2)	7 (4.6)	6 (3.9)	—
Partial response	101 (32.8)	55 (35.9)	46 (29.7)	—
Unspecified response	113 (36.7)	56 (36.6)	57 (36.8)	—
Stable disease	81 (26.3)	35 (22.9)	46 (29.7)	—

ECOG, Eastern Cooperative Oncology Group; IQR, interquartile range.

a Percentages were calculated excluding unknown values.

b Hemoglobin level of 10 g/dl is equivalent to 6.21 mmol/l.

c Percentages do not add to 100% because patients may have metastases at multiple localizations.

d This category includes patients who switched platinum-based regimens while receiving first-line chemotherapy.

### Treatments

About 64.3% of patients received immunotherapy following chemotherapy across the total cohort (Table 2). In the pembrolizumab era, 79 (51.6%) patients received pembrolizumab and 7 (4.6%) received avelumab. In the avelumab era, 15 (9.7%) received pembrolizumab and 97 (62.6%) received avelumab. The median time between completing chemotherapy and start of pembrolizumab was 154 days (IQR 112-240 days) in the pembrolizumab era, whereas in the avelumab era, the median time to start avelumab was 35 days (IQR 28-49 days).

**Table 2:** Descriptives of systemic treatment strategies

Descriptives	Total	Period of completing chemotherapy	
		Pembrolizumab era	Avelumab era
**Total, *n* (%)**	308 (100)	153 (100)	155 (100)
**Systemic therapy following chemotherapy, *n* (%)**			
Pembrolizumab	94 (30.5)	79 (51.6)	15 (9.7)
Avelumab^a^	104 (33.8)	7 (4.6)	97 (62.6)
Other (i.e. chemotherapy, other immunotherapy)	12 (3.9)	10 (6.5)	2 (1.3)
None	98 (31.8)	57 (37.3)	41 (26.5)
**Details of pembrolizumab treatment**^b^			
Ongoing treatment, *n* (%)	5 (5.3)	3 (3.8)	2 (13.3)
Median treatment duration (weeks), IQR	15 (6-54)	18 (7-59)	10 (3-15)
**Details of avelumab treatment**^b^			
Ongoing treatment, *n* (%)	36 (34.6)	—	36 (37.1)
Median treatment duration (weeks), IQR	16 (8-24)	32 (18-94)	14 (8-24)
**Third-line systemic therapy, *n* (%)**			
Chemotherapy	15 (4.9)	8 (5.2)	7 (4.5)
Immunotherapy^c^	15 (4.9)	9 (5.9)	6 (3.9)
Enfortumab vedotin	14 (4.5)	2 (1.3)	12 (7.7)
None	264 (85.7)	134 (87.6)	130 (83.9)

IQR, interquartile range.

a Avelumab was administered in the pembrolizumab era without reimbursement, for instance as part of a clinical study or other nonstandard care settings.

b Percentages based on the subset of patients who received pembrolizumab or avelumab.

c Including four patients with pembrolizumab rechallenge and two avelumab rechallenge [as part of a clinical study (AVE-SHORT)^25^].

In the pembrolizumab era, the median treatment duration and proportion still on treatment at data cut-off were 18 weeks (IQR 7-59 weeks) and 4% for patients receiving pembrolizumab, versus 32 weeks (IQR 18-94 weeks) and 0% for patients receiving avelumab. In the avelumab era, these were 10 weeks (IQR 3-15 weeks) and 13% for patients receiving pembrolizumab, versus 14 weeks (IQR 8-24 weeks) and 37% for patients receiving avelumab.

In both the pembrolizumab and avelumab eras, the majority of patients did not proceed to third-line treatment (e.g. enfortumab vedotin) following ICI (87.6% and 83.9%, respectively).

### Overall survival

The median (IQR) follow-up time among patients without events was 57.1 months (45.0-68.2 months) for OS in the pembrolizumab era and 15.7 months (11.8-23.9 months) for OS in the avelumab era.

**Figure 1:**
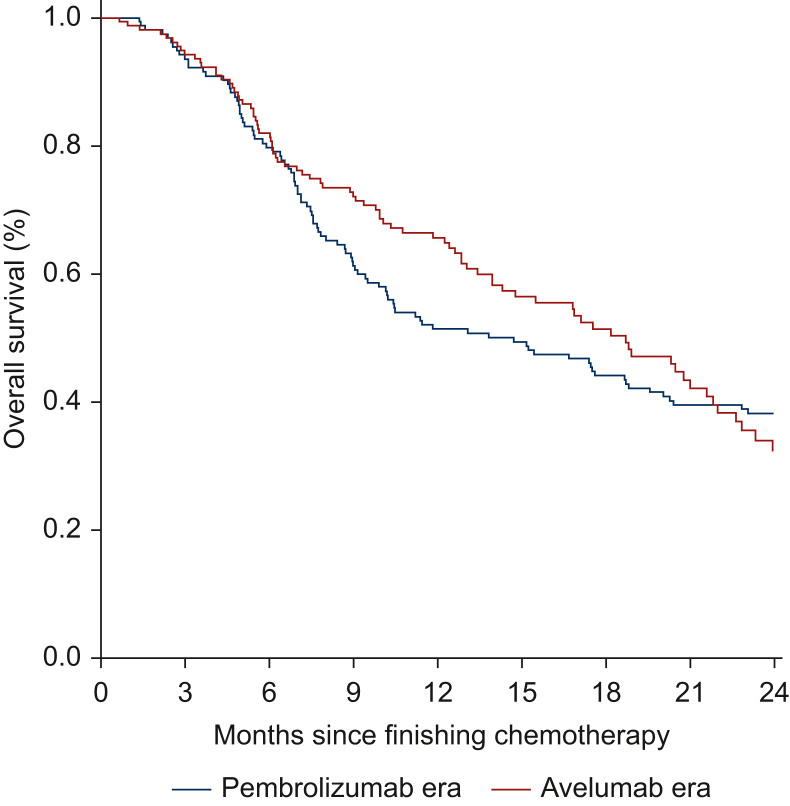
Unadjusted overall survival of patients with metastatic urothelial cancer of the bladder following platinum-based chemotherapy, stratified by periods with different immune checkpoint inhibitor standard of care.

Unadjusted median OS of patients completing 1L chemotherapy was 14.7 months (95% CI 10.2-20.1 months) and 18.7 months (95% CI 14.3-22.0 months) in the pembrolizumab era and avelumab era, respectively (Figure 1), with corresponding 2-year observed OS of 38% (95% CI 31% to 47%) and 32% (95% CI 24% to 43%). The median OS for ICI-treated patients in the pembrolizumab era and avelumab era were 18.1 months (95% CI 13.1 months-NE) and 18.2 months (95% CI 14.3-21.6 months), respectively.

**Figure 2:**
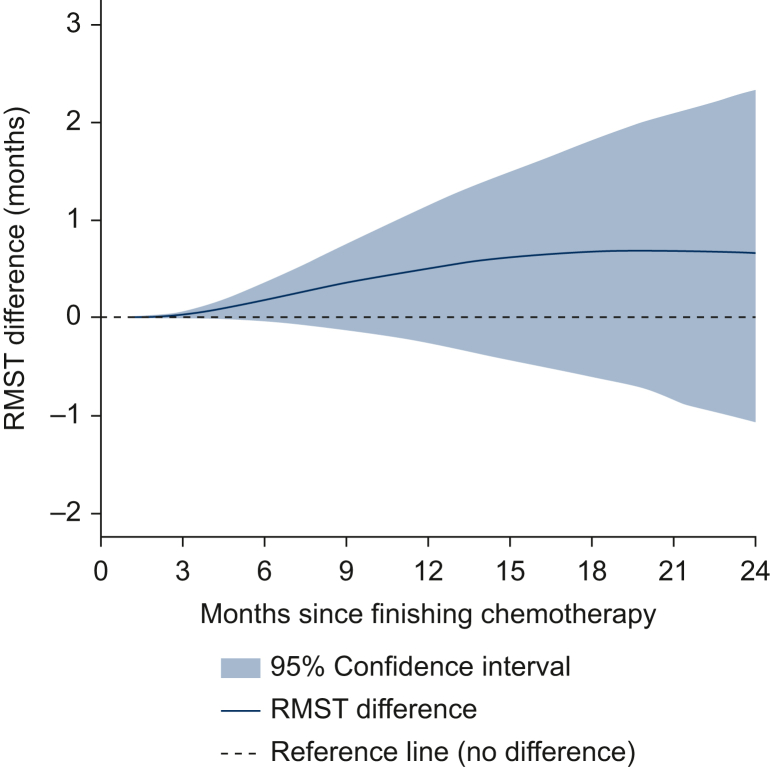
**Adjusted marginal restricted mean survival time (RMST) differences up to 24 months between patients who completed first-line platinum-based chemotherapy during the avelumab era and the pembrolizumab era.** A difference of >0 indicates a survival benefit for patients in the avelumab era; a difference of <0 indicates a survival benefit of patients treated in the pembrolizumab era.

Adjusted for sex, age, performance status, hemoglobin, 1L chemotherapy type and response, and metastatic sites (liver, bone, lung, lymph node, lymph node only and other), RMST up to 24 months was 15.6 months in the avelumab era and 14.9 months in the pembrolizumab era. RMST difference (avelumab era – pembrolizumab era) up to 24 months was 0.7 months (95% CI –1.1 to 2.3 months). RMST differences at all time points up to 24 months were not statistically different from 0 months (Figure 2). The adjusted time-varying HR for OS comparing avelumab era with pembrolizumab era is presented in Supplementary Figure S2, available at https://doi.org/10.1016/j.esmorw.2026.100685.

## Discussion

In this unique real-world population-based cohort, we assessed whether the change in standard of care after 1L platinum-based chemotherapy from pembrolizumab upon progression to avelumab maintenance treatment improved OS in mUCB patients. We observed no OS difference during the first 2 years after chemotherapy between patients who completed 1L chemotherapy in the avelumab era and those who completed 1L chemotherapy in the pembrolizumab era. These results indicate no survival benefit following the change in standard of care from pembrolizumab at progression to avelumab maintenance, while the ICI exposure in the avelumab era was higher.

No randomized trials have directly compared avelumab maintenance with pembrolizumab at progression following 1L platinum-based chemotherapy, due to partially overlapping timelines of the KEYNOTE-045 and JAVELIN Bladder-100 trials.^4^^,^^5^ A cross-trial comparison is not warranted due to key differences (e.g. inclusion criteria regarding number of chemotherapy cycles). Available observation studies comparing these treatment strategies were scarce and generally of poor quality, with considerable methodological differences to the current study. Earlier studies did not properly handle immortal time bias resulting from the different timings of both ICI applications, and sample sizes were generally very small. While acknowledging these limitations, other cohorts from Japan and Italy also demonstrated no difference in OS between patients who received avelumab or pembrolizumab after stable disease following 1L platinum-based chemotherapy.^7^^,^^8^^,^^10^

In this cohort study, we estimated the effect of different standards of care in the eligible population, but the observed effect is of course impacted by the proportion of actual use of both ICI strategies. In the pembrolizumab era, 56.2% received pembrolizumab or avelumab (51.6% and 4.6%, respectively) versus 72.3% in the avelumab era (9.7% and 62.6%, respectively). Under the assumption that both ICI treatments were equally effective, the mere application difference would result in an apparent benefit of the avelumab regimen. The fact that such a benefit was not observed, therefore, further strengthens the interpretation that the shift toward maintenance avelumab as standard of care did not yield a survival benefit. The observed proportions of patients receiving subsequent ICI were in line (e.g. a cohort from the United States) or somewhat higher than (e.g. a cohort from Spain) other estimates.^19^^,^^20^

The impact of both treatment strategies on quality of life is an important consideration.^21^ A treatment-free interval following intensive chemotherapy,^22^ which may be valuable to many patients, was eliminated or substantially shortened with the adoption of avelumab following chemotherapy. In addition, avelumab is administered more frequently than pembrolizumab (Q2W versus Q3W or Q6W, respectively), hence requiring more frequent hospital visits. Available data on toxicity profiles associated with each ICI do not allow a direct comparison. However, the higher proportion of patients receiving ICI in the avelumab era results in more patients being at risk for treatment-associated toxicities, which may negatively impact their quality of life. The potential harms and burdens of each treatment strategy should be carefully weighed against the apparent effectiveness. However, the current study cannot rule out a potential OS advantage for a subgroup of patients with a more favorable prognosis (e.g. patients with lymph node-only disease) where the potential disadvantages would be outweighed.

The findings of this study should be interpreted in light of several strengths and limitations. First, we consider the data source a major strength, using real-world data from a population-based nationwide cohort of mUCB patients in the Netherlands. The ProBCI database, embedded within the NCR, provides a unique infrastructure for patient follow-up across all hospitals in the Netherlands, ensuring complete survival data. The study is further strengthened by methodological choices. To minimize selection bias, we considered two standard of care periods, namely, pembrolizumab (till 2022) and avelumab (since 2022), instead of selecting patients based on received treatment. Although we cannot entirely rule out the possibility of other differences between the periods that may have affected survival, the similarity in baseline characteristics, combined with adjustment for these characteristics and the very minimal differences in subsequent treatments, provide confidence that confounding is unlikely to have significantly affected our results. In addition, we used a parametric survival model to estimate the difference in RMST, providing an absolute and clinically interpretable measure of OS difference that can be better weighed against possible disadvantages.

The main limitation of the study concerns data completeness, resulting from the retrospective nature of the study. A limited number of variables, such as performance status, were not available for all patients as data were derived from electronic health records; this was handled by using a missingness category in the analysis. Moreover, the OS effect could not be estimated over a period longer than 2 years after completing chemotherapy, due to the recency of the introduction of avelumab in the Netherlands in 2022. However, we expect this to have minimal effect on the outcomes as median OS and PFS in mUC are considerably shorter than 2 years.^5^ In addition, the cohort did not include patients with upper tract urothelial carcinoma because these are not covered in the ProBCI data, which means that findings only apply to patients with mUCB. Lastly, another potential limitation concerns the sample size, with a larger sample size allowing for more precise OS estimates. However, the present study is larger than comparable studies and the CI was sufficiently narrow to exclude an OS difference of clinically relevant magnitude.

The treatment landscape for mUCB is currently changing, with enfortumab vedotin plus pembrolizumab emerging as a 1L treatment option, based on the findings of the EV-302/KEYNOTE 39A trial.^23^ Until recently, 1L platinum-based chemotherapy followed by maintenance avelumab or alternatively pembrolizumab upon disease progression was the only available sequence. However, this approach will still be the treatment of choice for a subgroup of patients (e.g. due to contraindications for or unavailability of the new regimen).^3^^,^^23^^,^^24^

### Conclusions

This study demonstrated that for patients with mUCB who were progression free after at least four cycles of platinum-based chemotherapy, pembrolizumab at progression yielded similar OS as immediate maintenance avelumab, while minimizing ICI exposure. These findings suggest that a treatment-free interval after chemotherapy, followed by ICI at progression may be as effective as immediate maintenance ICI therapy in terms of OS. In the absence of strong randomized controlled trial evidence and based on these findings, the guidelines committee could consider a more balanced recommendation for either treatment strategy.
