# Supplementary material for: Real-world comparison of avelumab maintenance versus pembrolizumab at progression after first-line platinum-based chemotherapy in metastatic urothelial cancer
Source: ESMO Real World Data Digit Oncol. 2026 Feb 16;11:100685. doi: 10.1016/j.esmorw.2026.100685 (PMC13040931; doi:10.1016/j.esmorw.2026.100685)
Supplement: Supplementary Material [file mmc1.docx]

**Supplementary material**

**Supplementary figure 1: Flowchart of patient selection**

Patients who did not receive first-line systemic treatment (n=1,036)

Patients who received treatment in foreign hospital (n=7)

Patients who received first-line immuno- or immunochemotherapy (n=84)

Patients receiving other systemic therapy (n=22)

All new diagnoses of synchronous metastatic urothelial bladder cancer in the Netherlands in

Nov 1, 2017 – Dec 31, 2023

N=1,859

Patients who received ≥ 4 cycles of platinum-based chemotherapy

N=422

Patients who did receive < 4 cycles of platinum-based chemotherapy (n=282)

Unknown number of cycles/ongoing treatment (n=6)

Patients who received platinum- based first-line chemotherapy

N=710

Patients with disease progression during or within 42 days after completing chemotherapy (n=94)

Unknown response to chemotherapy (n=12)

Patients who died within 30 days after chemotherapy (n=8)

Patients without disease progression after minimal 4 cycles of platinum-based chemotherapy

**(Final cohort)**

N=308

**Completed chemotherapy after**

**01-01-2022**

**Avelumab era group**

N=155

**Completed chemotherapy on or before 01-01-2022**

**Pembrolizumab era group**

N=153

**Supplementary figure 2: Adjusted time-varying (conditional) Hazard Ratios (HR) for overall survival comparing avelumab era versus pembrolizumab era**

**
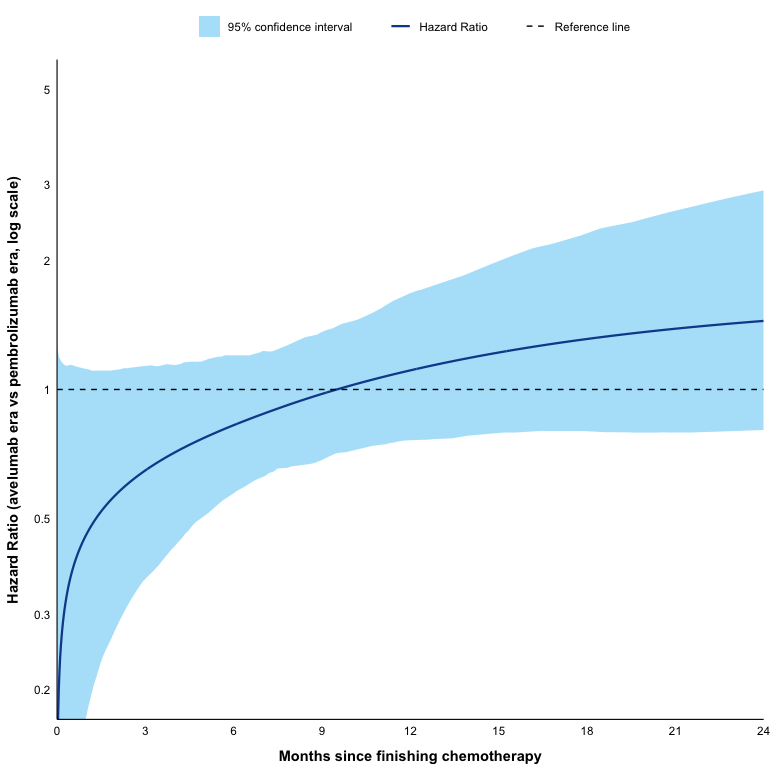
**

The figure illustrates the time-varying hazard ratio adjusted for sex, age, performance status, hemoglobin levels, first-line chemotherapy regimen, response to first-line chemotherapy and metastatic site (liver, bone, lung, lymph node only and other).
